# Supplementary material for: The ketogenic diet alleviates autoimmune thyroiditis caused by Th17/Treg imbalance by inhibiting the HMGB1/NLRP3 signaling pathway
Source: PLoS One. 2026 May 8;21(5):e0341564. doi: 10.1371/journal.pone.0341564 (PMC13155659; doi:10.1371/journal.pone.0341564)
Supplement: S1 File — (PDF) [file pone.0341564.s001.pdf]

Fig.1. Ketogenic diet attenuates autoimmune thyroiditis induced by high iodine intake in mice.

(B) Serum TgAb and TPOAb levels .

TGAB		
WT+ND	AIT+ND	AIT+KD
2.293154	4.460868	3.200249
3.331795	4.551767	2.691326
3.07543	3.984336	3.328657
3.253433	4.083608	3.968351
2.519245	4.099646	3.172125
3.003862	4.753896	3.796182

Tukey's multiple comparisons test	Mean Diff.	95.00% CI of diff.	Below threshold?	Summary	Adjusted P Value			
WT+ND vs. AIT+ND	-1.41	-2.011 to -0.8080	Yes	****	<0.0001	A-B		
WT+ND vs. AIT+KD	-0.4467	-1.048 to 0.1549	No	ns	0.1649	A-C		
AIT+ND vs. AIT+KD	0.9629	0.3613 to 1.564	Yes	**	0.0023	B-C		
Test details	Mean 1	Mean 2	Mean Diff.	SE of diff.	n1	n2	q	DF
WT+ND vs. AIT+ND	2.913	4.322	-1.41	0.2316	6	6	8.608	15
WT+ND vs. AIT+KD	2.913	3.359	-0.4467	0.2316	6	6	2.728	15
AIT+ND vs. AIT+KD	4.322	3.359	0.9629	0.2316	6	6	5.88	15

TPOAB		
WT+ND	AIT+ND	AIT+KD
22.230683	32.556894	21.901515
18.109664	70.488534	27.266964
9.852516	36.474801	22.757566
19.596211	30.567909	22.095836
17.028854	46.36347	27.671112
9.739206	33.998102	26.068408

Tukey's multiple comparisons test	Mean Diff.	95.00% CI of diff.	Below threshold?	Summary	Adjusted P Value	
WT+ND vs. AIT+ND	-25.65	-39.69 to -11.61	Yes	***	0.0007	A-B
WT+ND vs. AIT+KD	-8.534	-22.57 to 5.505	No	ns	0.2847	A-C
AIT+ND vs. AIT+KD	17.11	3.076 to 31.15	Yes	*	0.0165	B-C

Test details	Mean 1	Mean 2	Mean Diff.	SE of diff.	n1	n2	q	DF
WT+ND vs. AIT+ND	16.09	41.74	-25.65	5.405	6	6	6.711	15
WT+ND vs. AIT+KD	16.09	24.63	-8.534	5.405	6	6	2.233	15
AIT+ND vs. AIT+KD	41.74	24.63	17.11	5.405	6	6	4.478	15

(D) lymphocyte infiltration ratingand (E) score .

Infiltration Index		
WT+ND	AIT+ND	AIT+KD
1	3	1
2	3	2
2	3	2
2	4	2
1	2	1
1	3	2
1	4	2
2	3	3
1	4	2
1	3	2

Tukey's multiple comparisons test	Mean Diff.	95.00% CI of diff.	Below threshold?	Summary	Adjusted P Value	
WT+ND vs. AIT+ND	-1.8	-2.437 to -1.163	Yes	****	<0.0001	A-B
WT+ND vs. AIT+KD	-0.5	-1.137 to 0.1366	No	ns	0.145	A-C
AIT+ND vs. AIT+KD	1.3	0.6634 to 1.937	Yes	****	<0.0001	B-C

Test details	Mean 1	Mean 2	Mean Diff.	SE of diff.	n1	n2	q	DF
WT+ND vs. AIT+ND	1.4	3.2	-1.8	0.2568	10	10	9.914	27
WT+ND vs. AIT+KD	1.4	1.9	-0.5	0.2568	10	10	2.754	27
AIT+ND vs. AIT+KD	3.2	1.9	1.3	0.2568	10	10	7.16	27

Lymphocyte penetration index (score)		
WT+ND	AIT+ND	AIT+KD
1	3	1
2	3	2
2	3	2
2	4	2
1	2	1
1	3	2
1	4	2
2	3	1
1	4	2
1	3	2
1	3	1
2	3	2
2	3	2
2	4	2
1	2	1
1	3	2
1	4	2
2	3	1
1	4	2
1	3	2

Tukey's multiple comparisons test	Mean Diff.	95.00% CI of diff.	Below threshold?	Summary	Adjusted P Value	
WT+ND vs. AIT+ND	-1.8	-2.206 to -1.394	Yes	****	<0.0001	A-B
WT+ND vs. AIT+KD	-0.3	-0.7057 to 0.1057	No	ns	0.1856	A-C
AIT+ND vs. AIT+KD	1.5	1.094 to 1.906	Yes	****	<0.0001	B-C

Test details	Mean 1	Mean 2	Mean Diff.	SE of diff.	n1	n2	q	DF
WT+ND vs. AIT+ND	1.4	3.2	-1.8	0.1686	20	20	15.1	57
WT+ND vs. AIT+KD	1.4	1.7	-0.3	0.1686	20	20	2.517	57
AIT+ND vs. AIT+KD	3.2	1.7	1.5	0.1686	20	20	12.58	57

(F) Body weight of mice was continuously monitored during the dietary intervention

	WT+ND																			
0	20.09	21.34	19.87	18.76	19.23	21.45	18.98	20.12	21.76	19.54	20.65	18.32	19.09	21.56	20.43	18.12	21.87	19.25	20.34	22
4	20.39	21.54	19.87	18.96	19.43	21.75	19.5	20.7	22.3	20.1	20.1	19.12	20.1	22.16	20.1	20.1	21.25	19.43	21.25	22.3
8	20.59	21.61	20.14	19.1	19.58	22	20	21.2	22.75	20.6	20.6	19.83	20.6	22.7	20.6	20.6	21.8	19.58	21.8	22.75
12	21.05	21.86	20.39	19.41	19.91	22.45	20.45	21.65	23.15	21.05	21.05	20.47	21.05	23.19	21.05	21.05	22.3	19.91	22.3	23.15
16	21.48	22.09	20.62	19.69	20.22	22.85	20.85	22.05	23.5	21.45	21.45	21.05	21.45	23.63	21.45	21.45	22.75	20.22	22.75	23.5
20	21.88	22.3	20.83	19.95	20.51	23.2	21.2	22.4	23.8	21.8	21.8	21.58	21.8	24.03	21.8	21.8	22.5	20.51	23.15	23.8
24	22.25	22.49	21.03	20.19	20.78	23.5	21.5	22.92	24.05	22.1	22.1	22.06	22.1	24.39	22.1	22.1	23.5	20.78	23.5	24.05
28	22.6	22.92	21.21	22.92	21.04	22.92	22.92	22.95	22.92	22.5	22.35	22.92	22.92	24.71	22.35	22.35	22.5	21.04	23.8	24.25
32	22.92	22.83	22.92	20.61	21.28	23.95	22	23.18	24.42	22.55	22.55	22.5	22.35	25	22.55	22.55	24.05	21.28	24.05	24.42
36	23.22	22.92	21.54	20.8	22.92	24.12	22.2	23.38	22.5	22.72	22.72	22.5	22.35	25.26	22.92	22.72	22.92	21.51	24.25	24.56
40	23.5	23.12	21.69	22.92	21.72	22.92	22.92	23.56	24.68	22.86	22.86	23.59	22.35	22.92	22.86	22.86	24.42	21.72	24.42	24.68
44	23.76	23.25	21.83	21.13	21.92	24.4	22.54	22.19	22.19	22.92	22.19	22.19	22.98	22.19	22.98	22.98	22.5	21.92	24.57	24.78
48	24	23.37	21.96	21.27	22.1	24.51	22.68	23.86	24.86	22.19	23.08	22.92	23.08	25.87	22.92	23.08	24.69	22.1	24.69	24.86
52	24.22	23.48	22.08	21.4	22.27	24.61	22.8	23.99	24.93	23.16	23.16	24.41	23.11	26.03	22.19	23.16	24.8	22.27	24.8	24.93
56	24.43	23.58	22.19	21.52	22.43	24.7	22.9	24.1	24.99	23.23	23.23	24.63	23.23	23.11	23.23	23.23	24.89	22.43	24.89	24.99
60	24.62	23.67	22.29	21.63	22.58	24.78	22.98	24.2	23.11	23.29	22.92	24.83	23.29	26.3	23.29	23.29	24.96	22.58	24.96	25.04
64	24.8	23.75	23.11	23.11	22.72	24.85	23.05	24.29	25.08	23.34	23.34	25.01	23.34	22.92	22.92	23.34	25.02	22.72	25.02	25.08
68	24.96	23.82	22.19	21.82	22.85	24.91	23.11	24.37	23.11	23.38	23.11	25.17	23.11	26.51	23.38	23.38	25.07	22.85	25.07	25.11
72	25.11	23.11	22.55	22.9	22.97	24.96	23.16	24.44	25.14	23.42	23.42	25.32	23.42	26.6	23.42	23.42	23.11	23.11	25.11	25.14
76	25.25	23.95	22.62	22.97	23.08	25.01	23.2	24.5	25.16	23.45	23.45	25.45	23.45	26.68	23.45	23.45	25.15	23.08	25.15	25.16
80	25.38	24	23.69	23.04	23.19	25.05	23.23	24.55	25.18	23.48	23.48	25.57	23.48	26.75	23.48	23.11	25.18	23.19	25.18	25.18
84	25.5	24.05	24.75	24.1	24.5	24.99	24.5	24.99	24.99	24.5	24.5	24.99	24.5	24.99	24.5	24.5	24.99	24.5	24.99	24.99

	AIT+ND																			
0	18.78	21.12	19.98	18.45	20.76	21.34	19.56	20.89	18.23	21.67	20.54	19.43	21.09	18.67	20.12	21.98	19.23	18.87	20.78	21.54
4	20.85	19.62	21.63	22.37	21.42	21.62	20.83	19.37	21.61	22.2	20.76	21.34	19.56	20.89	20.69	22.53	20.08	19.72	21.38	22.1
8	20.63	21.85	19.58	20.66	20.61	21.8	21.2	22.51	20.31	22.95	21.61	22.19	20.44	21.77	21.19	22.98	20.8	20.45	21.9	22.59
12	21.05	22.33	19.91	23.17	20.88	22.32	19.98	23.17	20.88	23.52	19.98	18.45	20.76	21.34	19.56	20.89	21.42	21.09	22.36	23.03
16	21.45	22.75	20.22	23.76	21.36	21.62	20.83	23.76	21.36	24.06	20.83	19.37	21.61	22.19	20.44	20.67	20.66	21.86	19.58	23.42
20	21.86	22.5	20.51	21.84	21.82	22.54	21.51	24.29	21.78	24.54	21.51	20.16	22.31	22.9	21.2	21.05	21.05	22.37	19.91	22.31
24	20.16	22.31	22.9	22.1	22.11	23.51	20.78	24.77	22.15	22.43	22.08	20.86	22.92	23.52	21.88	21.45	21.45	22.75	20.22	22.92
28	20.86	22.92	23.52	22.35	22.35	22.58	21.04	25.21	22.49	22.78	22.56	21.48	23.45	24.06	22.48	21.86	21.82	22.55	20.51	23.45
32	21.48	23.45	24.06	22.55	22.55	24.05	21.28	25.61	22.8	23.1	22.97	22.04	23.92	24.54	22.99	22.19	22.16	23.54	20.78	23.92
36	22.04	23.92	24.54	22.92	22.72	22.92	21.51	25.98	23.08	23.39	23.33	22.54	24.34	24.97	23.46	22.35	22.35	22.59	21.04	24.34
40	22.54	24.34	24.97	22.86	22.86	24.42	21.72	26.32	23.34	23.66	23.65	22.99	24.72	25.36	23.89	22.55	22.55	24.05	24.6	24.72
44	22.99	24.72	25.36	22.98	22.98	22.59	25.3	26.63	23.58	23.91	23.94	23.39	25.06	25.71	24.29	22.92	22.72	22.92	21.51	25.06
48	23.39	25.06	25.71	22.92	23.08	22.8	22.19	26.92	23.8	24.14	24.2	23.76	25.37	26.03	24.66	22.86	22.86	24.7	21.72	25.37
52	23.76	25.37	26.03	23.16	23.16	24.88	22.27	27.19	23.99	24.35	24.44	24.1	25.65	26.32	24.99	22.98	22.98	22.56	21.92	25.65
56	24.1	25.65	26.32	23.23	23.23	24.89	22.43	27.44	24.17	24.55	24.66	24.41	25.91	26.59	25.3	22.92	23.08	25.2	22.11	25.91
60	24.41	25.91	26.59	25.3	23.29	24.96	22.58	27.67	24.33	24.73	24.86	24.69	26.14	26.83	25.59	23.16	25.2	24.82	25.6	26.14
64	24.69	26.14	26.83	25.59	26.92	23.8	26.55	27.88	24.48	24.9	25.05	24.95	26.35	27.05	25.86	23.23	23.23	24.89	22.43	26.35
68	24.95	26.35	27.05	25.86	27.19	23.99	26.75	28.07	24.62	25.06	25.22	25.19	26.54	27.25	26.11	23.29	23.29	24.96	22.58	26.54
72	25.19	26.54	27.25	26.11	27.44	24.17	26.93	28.25	24.74	25.21	25.38	25.41	26.71	27.43	26.34	27.67	25.64	25.98	25.41	26.71
76	25.41	26.71	27.43	26.34	27.67	24.33	27.1	28.31	24.85	25.35	25.53	25.61	26.87	27.59	26.55	27.88	25.77	26.14	25.61	26.87
80	25.61	26.87	27.59	26.55	27.88	24.48	27.25	28.46	24.95	25.48	25.67	25.8	27.01	27.74	26.75	28.07	25.88	26.29	26.3	26.75
84	26	26.18	26.5	26.5	26.8	26.9	26.7	27.34	26.22	25.6	25.8	25.97	27.14	27.87	26.93	28.25	27	27.04	27.18	27.47

	AIT+KD																			
0	19.65	20.45	18.34	21.23	19.78	20.98	21.12	19.98	18.45	20.76	21.34	19.56	20.89	18.78	21.65	20.56	19.45	20.32	21.9	18.23
4	20.08	20.85	19	21.6	22.3	21.42	21.62	20.83	19.37	21.61	22.19	20.44	21.77	20.78	19.95	20.96	19.9	20.72	22.15	18.78
8	20.66	20.6	21.88	19.58	22.51	20.31	22.9	21.2	22.51	20.31	22.9	21.2	22.51	21.26	20.69	21.29	20.28	21.06	22.38	19.25
12	20.8	21.05	22.39	19.91	23.17	20.88	21.12	19.98	20.31	20.88	23.52	21.88	23.17	21.69	21.35	21.57	20.61	21.35	22.58	19.66
16	21.1	21.45	22.75	20.8	23.76	21.36	21.62	20.83	20.88	21.36	24.06	20.16	22.31	22.9	21.2	22.51	20.31	21.61	22.76	20.02
20	21.87	21.87	22.51	20.51	24.29	21.78	22.05	21.51	21.36	21.78	24.54	20.86	22.92	23.52	21.88	23.17	20.88	21.84	22.92	20.35
24	22.12	22.18	23.5	20.78	24.77	22.15	22.43	22.08	21.78	22.15	24.97	21.48	23.45	24.06	22.48	23.76	21.36	22.05	23.07	20.64
28	22.35	22.35	22.56	21.04	25.21	22.49	22.78	22.56	22.15	22.49	25.36	22.04	23.92	24.54	22.99	24.29	21.78	22.24	23.2	20.91
32	22.55	22.55	24.05	21.28	25.61	22.86	23.19	22.97	22.49	22.8	25.71	22.54	24.34	24.97	23.46	24.77	22.15	22.41	23.32	21.15
36	22.92	22.72	22.2	21.51	25.98	23.08	23.39	23.33	22.88	23.08	26.03	22.99	24.72	25.36	23.89	25.21	22.49	22.57	23.43	21.37
40	22.86	22.86	24.42	21.72	24.1	23.34	23.66	23.65	23.08	23.34	26.32	23.39	25.06	25.71	24.29	25.61	22.8	22.71	23.53	21.57
44	22.98	22.98	22.51	21.92	26.63	23.58	23.91	25.3	23.34	23.58	26.59	23.76	25.37	26.03	24.66	25.98	23.08	22.84	23.62	21.76
48	22.92	23.08	24.69	22.11	26.92	23.89	24.14	24.23	23.58	23.8	26.83	24.1	25.65	26.32	24.99	26.32	23.34	22.96	23.7	21.93
52	23.16	23.5	24.82	22.27	27.19	23.99	24.35	24.44	23.89	23.99	27.05	24.41	25.91	26.59	25.3	26.63	23.58	23.07	23.78	22.09
56	23.23	23.23	24.89	22.43	27.44	24.17	24.55	24.66	23.99	24.17	27.25	24.69	26.14	26.83	25.59	26.92	23.8	23.17	23.85	22.24
60	23.29	23.29	24.96	23.8	27.67	24.33	24.73	24.86	24.17	24.33	27.43	24.95	26.35	27.05	25.86	27.19	23.99	23.26	23.91	22.38
64	25.59	26.92	23.8	26.55	27.88	24.48	24.94	25.05	24.33	24.48	27.59	25.19	26.54	27.25	26.11	27.44	24.17	23.34	23.97	22.51
68	25.86	27.19	23.99	26.75	28.07	24.62	25.06	25.22	23.8	27.01	27.74	25.41	26.71	27.43	26.34	27.67	24.33	23.41	24.02	22.63
72	27.43	26.34	27.67	24.33	26.35	27.05	25.86	27.19	23.99	27.14	27.87	25.61	26.87	27.59	26.55	27.88	24.48	23.48	24.07	22.74
76	23.3	26.55	24	24.48	26.54	27.25	26.11	27.44	24.17	27.26	27.99	27.1	28.31	25.37	26.79	23.35	23.04	23.54	24.11	22.84
80	23.36	24.16	24.12	25.41	26.71	27.43	26.34	27.67	24.33	27.37	28	27.25	28.46	25.49	26.95	23.39	23.1	23.6	24.15	22.94
84	25.57	25.55	25.58	25.61	26.87	27.59	26.55	27.88	24.48	26.8	26.9	26.7	27.34	25.5	26.88	24.88	24.33	24.7	24.76	24.58
