# Supplementary material for: The ketogenic diet alleviates autoimmune thyroiditis caused by Th17/Treg imbalance by inhibiting the HMGB1/NLRP3 signaling pathway
Source: PLoS One. 2026 May 8;21(5):e0341564. doi: 10.1371/journal.pone.0341564 (PMC13155659; doi:10.1371/journal.pone.0341564)
Supplement: S2 File — (PDF) [file pone.0341564.s002.pdf]

Fig.2. Effect of ketogenic diet on serum cytokines and oxidative stress markers in ND group.

(A) MCP-1

WT+ND	AIT+ND	AIT+KD
32.46	142.34	67.45
35.78	125.78	70.89
38.92	128.91	73.21
41.65	134.56	78.56
43.37	142.73	81.92
40.19	140.89	84.07

Tukey's multiple comparisons test	Mean Diff.	95.00% CI of diff.	Below threshold?	Summary	Adjusted P Value	
WT+ND vs. AIT+ND	-97.14	-106.3 to -87.98	Yes	****	<0.0001	A-B
WT+ND vs. AIT+KD	-37.29	-46.45 to -28.12	Yes	****	<0.0001	A-C
AIT+ND vs. AIT+KD	59.85	50.69 to 69.02	Yes	****	<0.0001	B-C

Test details	Mean 1	Mean 2	Mean Diff.	SE of diff.	n1	n2	q	DF
WT+ND vs. AIT+ND	38.73	135.9	-97.14	3.528	6	6	38.94	15
WT+ND vs. AIT+KD	38.73	76.02	-37.29	3.528	6	6	14.95	15
AIT+ND vs. AIT+KD	135.9	76.02	59.85	3.528	6	6	23.99	15

(B) TNF-α

WT+ND	AIT+ND	AIT+KD
271.23	412.78	365.12
294.56	456.34	378.45
317.89	498.56	390.78
330.12	512.78	402.34
352.45	543.9	415.67
378.78	576.23	428.9

Tukey's multiple comparisons test	Mean Diff.	95.00% CI of diff.	Below threshold?	Summary	Adjusted P Value	
WT+ND vs. AIT+ND	-175.9	-240.4 to -111.4	Yes	****	<0.0001	A-B
WT+ND vs. AIT+KD	-72.7	-137.2 to -8.206	Yes	*	0.0265	A-C
AIT+ND vs. AIT+KD	103.2	38.72 to 167.7	Yes	**	0.0023	B-C

Test details	Mean 1	Mean 2	Mean Diff.	SE of diff.	n1	n2	q	DF
WT+ND vs. AIT+ND	324.2	500.1	-175.9	24.83	6	6	10.02	15
WT+ND vs. AIT+KD	324.2	396.9	-72.7	24.83	6	6	4.141	15
AIT+ND vs. AIT+KD	500.1	396.9	103.2	24.83	6	6	5.879	15

(C)IL-1β

WT+ND	AIT+ND	AIT+KD
23.14	45.12	34.12
25.68	48.34	38.45
27.45	52.78	48.78
29.32	56.45	47.34
31.87	61.9	49.67
33.56	58.23	45.9

Tukey's multiple comparisons test	Mean Diff.	95.00% CI of diff.	Below threshold?	Summary	Adjusted P Value	
WT+ND vs. AIT+ND	-25.3	-33.71 to -16.89	Yes	****	<0.0001	A-B
WT+ND vs. AIT+KD	-15.54	-23.95 to -7.127	Yes	***	0.0006	A-C
AIT+ND vs. AIT+KD	9.76	1.347 to 18.17	Yes	*	0.0224	B-C

Test details	Mean 1	Mean 2	Mean Diff.	SE of diff.	n1	n2	q	DF
WT+ND vs. AIT+ND	28.5	53.8	-25.3	3.239	6	6	11.05	15
WT+ND vs. AIT+KD	28.5	44.04	-15.54	3.239	6	6	6.786	15
AIT+ND vs. AIT+KD	53.8	44.04	9.76	3.239	6	6	4.262	15

(D) IL-6

WT+ND	AIT+ND	AIT+KD
24.15	55.12	44.15
26.78	50.45	48.23
29.34	68.76	52.47
31.56	53.34	46.81
33.92	69.65	40.96
35.87	75.89	55.34

Tukey's multiple comparisons test	Mean Diff.	95.00% CI of diff.	Below threshold?	Summary	Adjusted P Value	
WT+ND vs. AIT+ND	-31.93	-42.81 to -21.05	Yes	****	<0.0001	A-B
WT+ND vs. AIT+KD	-17.72	-28.60 to -6.846	Yes	**	0.002	A-C
AIT+ND vs. AIT+KD	14.21	3.331 to 25.09	Yes	*	0.0105	B-C

Test details	Mean 1	Mean 2	Mean Diff.	SE of diff.	n1	n2	q	DF
WT+ND vs. AIT+ND	30.27	62.2	-31.93	4.188	6	6	10.78	15
WT+ND vs. AIT+KD	30.27	47.99	-17.72	4.188	6	6	5.985	15
AIT+ND vs. AIT+KD	62.2	47.99	14.21	4.188	6	6	4.798	15

(E) IL-18

WT+ND	AIT+ND	AIT+KD
29.15	54.15	34.23
36.89	48.23	46.78
29.47	42.47	39.45
32.13	56.81	42.12
34.65	61.35	47.89
36.98	55.99	54.32

Tukey's multiple comparisons test	Mean Diff.	95.00% CI of diff.	Below threshold?	Summary	Adjusted P Value	
WT+ND vs. AIT+ND	-19.96	-28.94 to -10.97	Yes	***	0.0001	A-B
WT+ND vs. AIT+KD	-10.92	-19.90 to -1.936	Yes	*	0.0169	A-C
AIT+ND vs. AIT+KD	9.035	0.05058 to 18.02	Yes	*	0.0486	B-C

Test details	Mean 1	Mean 2	Mean Diff.	SE of diff.	n1	n2	q	DF
WT+ND vs. AIT+ND	33.21	53.17	-19.96	3.459	6	6	8.159	15
WT+ND vs. AIT+KD	33.21	44.13	-10.92	3.459	6	6	4.465	15
AIT+ND vs. AIT+KD	53.17	44.13	9.035	3.459	6	6	3.694	15

(F) IFN-γ

WT+ND	AIT+ND	AIT+KD
34.12	94.56	64.12
25.67	97.89	45.45
26.89	100.23	66.78
27.34	103.56	58.01
28.56	108.78	69.34
29.78	112.45	72.67

Tukey's multiple comparisons test	Mean Diff.	95.00% CI of diff.	Below threshold?	Summary	Adjusted P Value	
WT+ND vs. AIT+ND	-74.19	-84.82 to -63.55	Yes	****	<0.0001	A-B
WT+ND vs. AIT+KD	-34	-44.64 to -23.37	Yes	****	<0.0001	A-C
AIT+ND vs. AIT+KD	40.18	29.55 to 50.82	Yes	****	<0.0001	B-C

Test details	Mean 1	Mean 2	Mean Diff.	SE of diff.	n1	n2	q	DF
WT+ND vs. AIT+ND	28.73	102.9	-74.19	4.095	6	6	25.62	15
WT+ND vs. AIT+KD	28.73	62.73	-34	4.095	6	6	11.74	15
AIT+ND vs. AIT+KD	102.9	62.73	40.18	4.095	6	6	13.88	15

(G) MDA

WT+ND	AIT+ND	AIT+KD
34.12	56.12	44.12
36.58	59.45	46.78
38.74	61.78	49.35
40.91	64.32	51.67
43.27	68.55	53.21
44.63	72.9	54.89

Tukey's multiple comparisons test	Mean Diff.	95.00% CI of diff.	Below threshold?	Summary	Adjusted P Value	
WT+ND vs. AIT+ND	-24.15	-31.39 to -16.90	Yes	****	<0.0001	A-B
WT+ND vs. AIT+KD	-10.3	-17.54 to -3.048	Yes	**	0.0058	A-C
AIT+ND vs. AIT+KD	13.85	6.603 to 21.10	Yes	***	0.0005	B-C

Test details	Mean 1	Mean 2	Mean Diff.	SE of diff.	n1	n2	q	DF
WT+ND vs. AIT+ND	39.71	63.85	-24.15	2.79	6	6	12.24	15
WT+ND vs. AIT+KD	39.71	50	-10.3	2.79	6	6	5.218	15
AIT+ND vs. AIT+KD	63.85	50	13.85	2.79	6	6	7.02	15

(H) SOD

WT+ND	AIT+ND	AIT+KD
101.23	62.45	91.23
105.67	67.89	93.45
109.87	71.23	96.78
113.45	75.67	99.01
118.76	80.98	102.34
124.32	84.56	104.56

Tukey's multiple comparisons test	Mean Diff.	95.00% CI of diff.	Below threshold?	Summary	Adjusted P Value	
WT+ND vs. AIT+ND	38.42	27.25 to 49.59	Yes	****	<0.0001	A-B
WT+ND vs. AIT+KD	14.32	3.153 to 25.49	Yes	*	0.0119	A-C
AIT+ND vs. AIT+KD	-24.1	-35.27 to -12.93	Yes	***	0.0001	B-C

Test details	Mean 1	Mean 2	Mean Diff.	SE of diff.	n1	n2	q	DF
WT+ND vs. AIT+ND	112.2	73.8	38.42	4.3	6	6	12.64	15
WT+ND vs. AIT+KD	112.2	97.9	14.32	4.3	6	6	4.711	15
AIT+ND vs. AIT+KD	73.8	97.9	-24.1	4.3	6	6	7.926	15

(I) T-AOC

WT+ND	AIT+ND	AIT+KD
8.12	3.12	8.12
8.45	3.45	8.45
9.78	3.78	9.78
10.34	4.23	10.34
11.67	4.67	11.56
12.9	4.9	9.89

Tukey's multiple comparisons test	Mean Diff.	95.00% CI of diff.	Below threshold?	Summary	Adjusted P Value	
WT+ND vs. AIT+ND	6.185	4.157 to 8.213	Yes	****	<0.0001	A-B
WT+ND vs. AIT+KD	0.52	-1.508 to 2.548	No	ns	0.7864	A-C
AIT+ND vs. AIT+KD	-5.665	-7.693 to -3.637	Yes	****	<0.0001	B-C

Test details	Mean 1	Mean 2	Mean Diff.	SE of diff.	n1	n2	q	DF
WT+ND vs. AIT+ND	10.21	4.025	6.185	0.7809	6	6	11.2	15
WT+ND vs. AIT+KD	10.21	9.69	0.52	0.7809	6	6	0.9418	15
AIT+ND vs. AIT+KD	4.025	9.69	-5.665	0.7809	6	6	10.26	15
