# Supplementary material for: The ketogenic diet alleviates autoimmune thyroiditis caused by Th17/Treg imbalance by inhibiting the HMGB1/NLRP3 signaling pathway
Source: PLoS One. 2026 May 8;21(5):e0341564. doi: 10.1371/journal.pone.0341564 (PMC13155659; doi:10.1371/journal.pone.0341564)
Supplement: S3 File — (PDF) [file pone.0341564.s003.pdf]

Fig.3. KD Decreased the Expression of HMGB1, TLR2, TLR4, NF-κB.

(A) Expression levels of protein in thyroid tissues of mice in each group.

HMGB1		
WT+ND	AIT+ND	AIT+KD
0.845495342	1.29482455	0.855976927
0.809107109	1.316561059	0.869386187
0.823763538	1.274747005	0.847373818

Tukey's multiple comparisons test	Mean Diff.	95.00% CI of diff.	Below threshold?	Summary	Adjusted P Value	
WT+ND vs. AIT+ND	-0.4693	-0.5125 to -0.4260	Yes	****	<0.0001	A-B
WT+ND vs. AIT+KD	-0.03146	-0.07474 to 0.01183	No	ns	0.1441	A-C
AIT+ND vs. AIT+KD	0.4378	0.3945 to 0.4811	Yes	****	<0.0001	B-C

Test details	Mean 1	Mean 2	Mean Diff.	SE of diff.	n1	n2	q	DF
WT+ND vs. AIT+ND	0.8261	1.295	-0.4693	0.01411	3	3	47.04	6
WT+ND vs. AIT+KD	0.8261	0.8576	-0.03146	0.01411	3	3	3.153	6
AIT+ND vs. AIT+KD	1.295	0.8576	0.4378	0.01411	3	3	43.89	6

TLR2

WT+ND	AIT+ND	AIT+KD
0.680705831	1.270973742	0.866473811
0.614669558	1.231384204	0.836959515
0.638222892	1.285603656	0.865492294

Tukey's multiple comparisons test	Mean Diff.	95.00% CI of diff.	Below threshold?	Summary	Adjusted P Value	
WT+ND vs. AIT+ND	−0.6181	−0.6858 to −0.5505	Yes	****	<0.0001	A-B
WT+ND vs. AIT+KD	−0.2118	−0.2794 to −0.1441	Yes	***	0.0002	A-C
AIT+ND vs. AIT+KD	0.4063	0.3387 to 0.4740	Yes	****	<0.0001	B-C

Test details	Mean 1	Mean 2	Mean Diff.	SE of diff.	n1	n2	q	DF
WT+ND vs. AIT+ND	0.6445	1.263	−0.6181	0.02205	3	3	39.64	6
WT+ND vs. AIT+KD	0.6445	0.8563	−0.2118	0.02205	3	3	13.58	6
AIT+ND vs. AIT+KD	1.263	0.8563	0.4063	0.02205	3	3	26.06	6

TLR4

WT+ND	AIT+ND	AIT+KD
0.941346397	1.292668968	1.068585329
0.977582244	1.334936679	1.108532451
1.041475992	1.382352045	1.12749157

Tukey's multiple comparisons test	Mean Diff.	95.00% CI of diff.	Below threshold?	Summary	Adjusted P Value	
WT+ND vs. AIT+ND	-0.3499	-0.4570 to -0.2427	Yes	***	0.0001	A-B
WT+ND vs. AIT+KD	-0.1147	-0.2219 to -0.007590	Yes	*	0.0384	A-C
AIT+ND vs. AIT+KD	0.2351	0.1280 to 0.3423	Yes	**	0.0013	B-C

Test details	Mean 1	Mean 2	Mean Diff.	SE of diff.	n1	n2	q	DF
WT+ND vs. AIT+ND	0.9868	1.337	-0.3499	0.03492	3	3	14.17	6
WT+ND vs. AIT+KD	0.9868	1.102	-0.1147	0.03492	3	3	4.647	6
AIT+ND vs. AIT+KD	1.337	1.102	0.2351	0.03492	3	3	9.522	6

NF-κB

WT+ND	AIT+ND	AIT+KD
0.635411594	1.249680442	0.967410476
0.702460373	1.295432958	1.023464328
0.71760734	1.298938284	1.007628154

Tukey's multiple comparisons test	Mean Diff.	95.00% CI of diff.	Below threshold?	Summary	Adjusted P Value	
WT+ND vs. AIT+ND	-0.5962	-0.6818 to -0.5106	Yes	****	<0.0001	A-B
WT+ND vs. AIT+KD	-0.3143	-0.4000 to -0.2287	Yes	****	<0.0001	A-C
AIT+ND vs. AIT+KD	0.2818	0.1962 to 0.3675	Yes	***	0.0001	B-C

Test details	Mean 1	Mean 2	Mean Diff.	SE of diff.	n1	n2	q	DF
WT+ND vs. AIT+ND	0.6852	1.281	-0.5962	0.0279	3	3	30.22	6
WT+ND vs. AIT+KD	0.6852	0.9995	-0.3143	0.0279	3	3	15.93	6
AIT+ND vs. AIT+KD	1.281	0.9995	0.2818	0.0279	3	3	14.28	6
