# Supplementary material for: The ketogenic diet alleviates autoimmune thyroiditis caused by Th17/Treg imbalance by inhibiting the HMGB1/NLRP3 signaling pathway
Source: PLoS One. 2026 May 8;21(5):e0341564. doi: 10.1371/journal.pone.0341564 (PMC13155659; doi:10.1371/journal.pone.0341564)
Supplement: S4 File — (PDF) [file pone.0341564.s004.pdf]

Fig.4. KD Decreased the Expression of NLRP3/ASC/Caspase-1 Signaling Pathway.

(A) Expression levels of NLRP3, ASC and Caspase-1 mRNA in thyroid tissues of mice in each group.

NLRP3		
WT+ND	AIT+ND	AIT+KD
1	2.39	0.45
1.01	2.45	0.66
1	2.33	0.67

Tukey's multiple comparisons test	Mean Diff.	95.00% CI of diff.	Below threshold?	Summary	Adjusted P Value	
WT+ND vs. AIT+ND	-1.387	-1.574 to -1.199	Yes	****	<0.0001	A-B
WT+ND vs. AIT+KD	0.41	-0.003753 to 0.8238	No	ns	0.0509	A-C
AIT+ND vs. AIT+KD	1.797	1.320 to 2.273	Yes	**	0.0032	B-C

Test details	Mean 1	Mean 2	Mean Diff.	SE of diff.	n1	n2	q	DF
WT+ND vs. AIT+ND	1.003	2.39	-1.387	0.0318	3	3	61.67	2
WT+ND vs. AIT+KD	1.003	0.5933	0.41	0.07024	3	3	8.255	2
AIT+ND vs. AIT+KD	2.39	0.5933	1.797	0.0809	3	3	31.41	2

ASC

WT+ND	AIT+ND	AIT+KD
1.06	2.13	0.38
0.99	2.48	0.37
0.95	2.33	0.37

Tukey's multiple comparisons test	Mean Diff.	95.00% CI of diff.	Below threshold?	Summary	Adjusted P Value	
WT+ND vs. AIT+ND	-1.313	-2.054 to -0.5726	Yes	*	0.0165	A-B
WT+ND vs. AIT+KD	0.6267	0.4555 to 0.7978	Yes	**	0.0035	A-C
AIT+ND vs. AIT+KD	1.94	1.325 to 2.555	Yes	**	0.0051	B-C

Test details	Mean 1	Mean 2	Mean Diff.	SE of diff.	n1	n2	q	DF
WT+ND vs. AIT+ND	1	2.313	-1.313	0.1257	3	3	14.77	2
WT+ND vs. AIT+KD	1	0.3733	0.6267	0.02906	3	3	30.5	2
AIT+ND vs. AIT+KD	2.313	0.3733	1.94	0.1044	3	3	26.28	2

Caspase-1

WT+ND	AIT+ND	AIT+KD
0.94	2.04	0.65
0.95	2.38	0.62
1.11	2.16	0.67

Tukey's multiple comparisons test	Mean Diff.	95.00% CI of diff.	Below threshold?	Summary	Adjusted P Value	
WT+ND vs. AIT+ND	-1.193	-1.896 to -0.4911	Yes	*	0.0179	A-B
WT+ND vs. AIT+KD	0.3533	0.08916 to 0.6175	Yes	*	0.0286	A-C
AIT+ND vs. AIT+KD	1.547	0.8957 to 2.198	Yes	**	0.0092	B-C

Test details	Mean 1	Mean 2	Mean Diff.	SE of diff.	n1	n2	q	DF
WT+ND vs. AIT+ND	1	2.193	-1.193	0.1192	3	3	14.16	2
WT+ND vs. AIT+KD	1	0.6467	0.3533	0.04485	3	3	11.14	2
AIT+ND vs. AIT+KD	2.193	0.6467	1.547	0.1105	3	3	19.79	2

(B) Expression levels of NLRP3, ASC and Caspase-1 protein in thyroid tissues of mice in each group.

NLRP3

WT+ND	AIT+ND	AIT+KD
0.835046485	1.264949902	1.153292052
0.834641038	1.289474367	1.145102616
0.834641038	1.335602857	1.09854063

Tukey's multiple comparisons test	Mean Diff.	95.00% CI of diff.	Below threshold?	Summary	Adjusted P Value	
WT+ND vs. AIT+ND	-0.4619	-0.5291 to -0.3947	Yes	****	<0.0001	A-B
WT+ND vs. AIT+KD	-0.2975	-0.3647 to -0.2303	Yes	****	<0.0001	A-C
AIT+ND vs. AIT+KD	0.1644	0.09716 to 0.2316	Yes	***	0.0007	B-C

Test details	Mean 1	Mean 2	Mean Diff.	SE of diff.	n1	n2	q	DF
WT+ND vs. AIT+ND	0.8348	1.297	-0.4619	0.0219	3	3	29.82	6
WT+ND vs. AIT+KD	0.8348	1.132	-0.2975	0.0219	3	3	19.21	6
AIT+ND vs. AIT+KD	1.297	1.132	0.1644	0.0219	3	3	10.61	6

ASC		
WT+ND	AIT+ND	AIT+KD
0.623116756	1.390719257	0.739767976
0.637935465	1.418936349	0.732046726
0.640701761	1.443298699	0.713847796

Tukey's multiple comparisons test	Mean Diff.	95.00% CI of diff.	Below threshold?	Summary	Adjusted P Value	
WT+ND vs. AIT+ND	-0.7837	-0.8285 to -0.7389	Yes	****	<0.0001	A-B
WT+ND vs. AIT+KD	-0.09464	-0.1394 to -0.04985	Yes	**	0.0016	A-C
AIT+ND vs. AIT+KD	0.6891	0.6443 to 0.7339	Yes	****	<0.0001	B-C

Test details	Mean 1	Mean 2	Mean Diff.	SE of diff.	n1	n2	q	DF
WT+ND vs. AIT+ND	0.6339	1.418	-0.7837	0.0146	3	3	75.93	6
WT+ND vs. AIT+KD	0.6339	0.7286	-0.09464	0.0146	3	3	9.168	6
AIT+ND vs. AIT+KD	1.418	0.7286	0.6891	0.0146	3	3	66.76	6

Caspase-1

WT+ND	AIT+ND	AIT+KD
0.502644495	0.880576141	0.698074803
0.487233913	0.874790433	0.663838929
0.479965386	0.904659268	0.643364884

Tukey's multiple comparisons test	Mean Diff.	95.00% CI of diff.	Below threshold?	Summary	Adjusted P Value	
WT+ND vs. AIT+ND	−0.3967	−0.4458 to −0.3477	Yes	****	<0.0001	A-B
WT+ND vs. AIT+KD	−0.1785	−0.2275 to −0.1294	Yes	****	<0.0001	A-C
AIT+ND vs. AIT+KD	0.2182	0.1692 to 0.2673	Yes	****	<0.0001	B-C

Test details	Mean 1	Mean 2	Mean Diff.	SE of diff.	n1	n2	q	DF
WT+ND vs. AIT+ND	0.4899	0.8867	−0.3967	0.01598	3	3	35.11	6
WT+ND vs. AIT+KD	0.4899	0.6684	−0.1785	0.01598	3	3	15.8	6
AIT+ND vs. AIT+KD	0.8867	0.6684	0.2182	0.01598	3	3	19.31	6
