# Supplementary material for: The ketogenic diet alleviates autoimmune thyroiditis caused by Th17/Treg imbalance by inhibiting the HMGB1/NLRP3 signaling pathway
Source: PLoS One. 2026 May 8;21(5):e0341564. doi: 10.1371/journal.pone.0341564 (PMC13155659; doi:10.1371/journal.pone.0341564)
Supplement: S5 File — (PDF) [file pone.0341564.s005.pdf]

Fig.5. KD Rescued Th17/Treg Imbalance in mice.

(A) Expression levels of RORγt, IL-17, FoxP3, IL-10 mRNA in thyroid tissues of mice in each group.

RORγt		
WT+ND	AIT+ND	AIT+KD
0.95	2.1	0.25
1.1	2.43	0.23
0.95	2.16	0.27

Tukey's multiple comparisons test	Mean Diff.	95.00% CI of diff.	Below threshold?	Summary	Adjusted P Value	
WT+ND vs. AIT+ND	-1.23	-1.542 to -0.9183	Yes	**	0.0028	A-B
WT+ND vs. AIT+KD	0.75	0.3949 to 1.105	Yes	*	0.0117	A-C
AIT+ND vs. AIT+KD	1.98	1.328 to 2.632	Yes	**	0.0056	B-C

Test details	Mean 1	Mean 2	Mean Diff.	SE of diff.	n1	n2	q	DF
WT+ND vs. AIT+ND	1	2.23	-1.23	0.05292	3	3	32.87	2
WT+ND vs. AIT+KD	1	0.25	0.75	0.06028	3	3	17.6	2
AIT+ND vs. AIT+KD	2.23	0.25	1.98	0.1106	3	3	25.32	2

IL-17

WT+ND	AIT+ND	AIT+KD
1.04	2.1	0.57
0.97	2.5	0.63
0.98	2.36	0.58

Tukey's multiple comparisons test	Mean Diff.	95.00% CI of diff.	Below threshold?	Summary	Adjusted P Value	
WT+ND vs. AIT+ND	−1.323	−2.140 to −0.5069	Yes	*	0.0196	A-B
WT+ND vs. AIT+KD	0.4033	0.1820 to 0.6246	Yes	*	0.0156	A-C
AIT+ND vs. AIT+KD	1.727	1.128 to 2.326	Yes	**	0.0062	B-C

Test details	Mean 1	Mean 2	Mean Diff.	SE of diff.	n1	n2	q	DF
WT+ND vs. AIT+ND	0.9967	2.32	−1.323	0.1386	3	3	13.5	2
WT+ND vs. AIT+KD	0.9967	0.5933	0.4033	0.03756	3	3	15.18	2
AIT+ND vs. AIT+KD	2.32	0.5933	1.727	0.1017	3	3	24.01	2

FoxP3

WT+ND	AIT+ND	AIT+KD
1.6	0.64	0.91
1.72	0.67	1.03
1.68	0.6	1.07

Tukey's multiple comparisons test	Mean Diff.	95.00% CI of diff.	Below threshold?	Summary	Adjusted P Value	
WT+ND vs. AIT+ND	1.03	0.8176 to 1.242	Yes	**	0.0013	A-B
WT+ND vs. AIT+KD	0.6633	0.5062 to 0.8204	Yes	**	0.0022	A-C
AIT+ND vs. AIT+KD	-0.3667	-0.7073 to -0.02600	Yes	*	0.0435	B-C

Test details	Mean 1	Mean 2	Mean Diff.	SE of diff.	n1	n2	q	DF
WT+ND vs. AIT+ND	1.667	0.6367	1.03	0.03606	3	3	40.4	2
WT+ND vs. AIT+KD	1.667	1.003	0.6633	0.02667	3	3	35.18	2
AIT+ND vs. AIT+KD	0.6367	1.003	-0.3667	0.05783	3	3	8.967	2

IL-10

WT+ND	AIT+ND	AIT+KD
2.11	0.5	1.01
2.23	0.69	1.03
2.35	0.66	0.97

Tukey's multiple comparisons test	Mean Diff.	95.00% CI of diff.	Below threshold?	Summary	Adjusted P Value	
WT+ND vs. AIT+ND	1.613	1.358 to 1.869	Yes	***	0.0002	A-B
WT+ND vs. AIT+KD	1.227	0.7441 to 1.709	Yes	**	0.0081	A-C
AIT+ND vs. AIT+KD	-0.3867	-0.7535 to -0.01984	Yes	*	0.0452	B-C

Test details	Mean 1	Mean 2	Mean Diff.	SE of diff.	n1	n2	q	DF
WT+ND vs. AIT+ND	2.23	0.6167	1.613	0.04333	3	3	52.65	2
WT+ND vs. AIT+KD	2.23	1.003	1.227	0.08192	3	3	21.18	2
AIT+ND vs. AIT+KD	0.6167	1.003	-0.3867	0.06227	3	3	8.781	2

(B)Expression levels of ROR γ t, IL-17, FoxP3, IL-10 protein in thyroid tissues of mice in each group

RORγt		
WT+ND	AIT+ND	AIT+KD
0.350645198	1.063448263	0.911450056
0.349137261	1.069408447	0.910081607
0.354220286	1.067938393	0.912962611

Tukey's multiple comparisons test	Mean Diff.	95.00% CI of diff.	Below threshold?	Summary	Adjusted P Value	
WT+ND vs. AIT+ND	-0.7156	-0.7218 to -0.7094	Yes	****	<0.0001	A-B
WT+ND vs. AIT+KD	-0.5602	-0.5664 to -0.5539	Yes	****	<0.0001	A-C
AIT+ND vs. AIT+KD	0.1554	0.1492 to 0.1617	Yes	****	<0.0001	B-C

Test details	Mean 1	Mean 2	Mean Diff.	SE of diff.	n1	n2	q	DF
WT+ND vs. AIT+ND	0.3513	1.067	-0.7156	0.002029	3	3	498.7	6
WT+ND vs. AIT+KD	0.3513	0.9115	-0.5602	0.002029	3	3	390.4	6
AIT+ND vs. AIT+KD	1.067	0.9115	0.1554	0.002029	3	3	108.3	6

IL-17

WT+ND	AIT+ND	AIT+KD
0.343372169	0.931622403	0.584447006
0.345152311	0.924906739	0.591586617
0.344230666	0.928241057	0.592847383

Tukey's multiple comparisons test	Mean Diff.	95.00% CI of diff.	Below threshold?	Summary	Adjusted P Value	
WT+ND vs. AIT+ND	-0.584	-0.5923 to -0.5757	Yes	****	<0.0001	A-B
WT+ND vs. AIT+KD	-0.2454	-0.2536 to -0.2371	Yes	****	<0.0001	A-C
AIT+ND vs. AIT+KD	0.3386	0.3304 to 0.3469	Yes	****	<0.0001	B-C

Test details	Mean 1	Mean 2	Mean Diff.	SE of diff.	n1	n2	q	DF
WT+ND vs. AIT+ND	0.3443	0.9283	-0.584	0.002691	3	3	306.9	6
WT+ND vs. AIT+KD	0.3443	0.5896	-0.2454	0.002691	3	3	128.9	6
AIT+ND vs. AIT+KD	0.9283	0.5896	0.3386	0.002691	3	3	178	6

FoxP3

WT+ND	AIT+ND	AIT+KD
0.757759898	0.390445538	0.564708287
0.756435692	0.391381173	0.5646929
0.757288176	0.391036155	0.571246576

Tukey's multiple comparisons test	Mean Diff.	95.00% CI of diff.	Below threshold?	Summary	Adjusted P Value	
WT+ND vs. AIT+ND	0.3662	0.3606 to 0.3718	Yes	****	<0.0001	A-B
WT+ND vs. AIT+KD	0.1903	0.1847 to 0.1959	Yes	****	<0.0001	A-C
AIT+ND vs. AIT+KD	-0.1759	-0.1815 to -0.1703	Yes	****	<0.0001	B-C

Test details	Mean 1	Mean 2	Mean Diff.	SE of diff.	n1	n2	q	DF
WT+ND vs. AIT+ND	0.7572	0.391	0.3662	0.001823	3	3	284.1	6
WT+ND vs. AIT+KD	0.7572	0.5669	0.1903	0.001823	3	3	147.6	6
AIT+ND vs. AIT+KD	0.391	0.5669	-0.1759	0.001823	3	3	136.5	6

IL-10

WT+ND	AIT+ND	AIT+KD
0.868810975	0.461811437	0.66565971
0.867052971	0.464852984	0.665333907
0.867384878	0.461654927	0.668383384

Tukey's multiple comparisons test	Mean Diff.	95.00% CI of diff.	Below threshold?	Summary	Adjusted P Value	
WT+ND vs. AIT+ND	0.405	0.4012 to 0.4088	Yes	****	<0.0001	A-B
WT+ND vs. AIT+KD	0.2013	0.1975 to 0.2051	Yes	****	<0.0001	A-C
AIT+ND vs. AIT+KD	-0.2037	-0.2075 to -0.1999	Yes	****	<0.0001	B-C

Test details	Mean 1	Mean 2	Mean Diff.	SE of diff.	n1	n2	q	DF
WT+ND vs. AIT+ND	0.8677	0.4628	0.405	0.001241	3	3	461.6	6
WT+ND vs. AIT+KD	0.8677	0.6665	0.2013	0.001241	3	3	229.4	6
AIT+ND vs. AIT+KD	0.4628	0.6665	-0.2037	0.001241	3	3	232.2	6
