# Supplementary figures and images for: The ketogenic diet alleviates autoimmune thyroiditis caused by Th17/Treg imbalance by inhibiting the HMGB1/NLRP3 signaling pathway
Source: PLoS One. 2026 May 8;21(5):e0341564. doi: 10.1371/journal.pone.0341564 (PMC13155659; doi:10.1371/journal.pone.0341564)

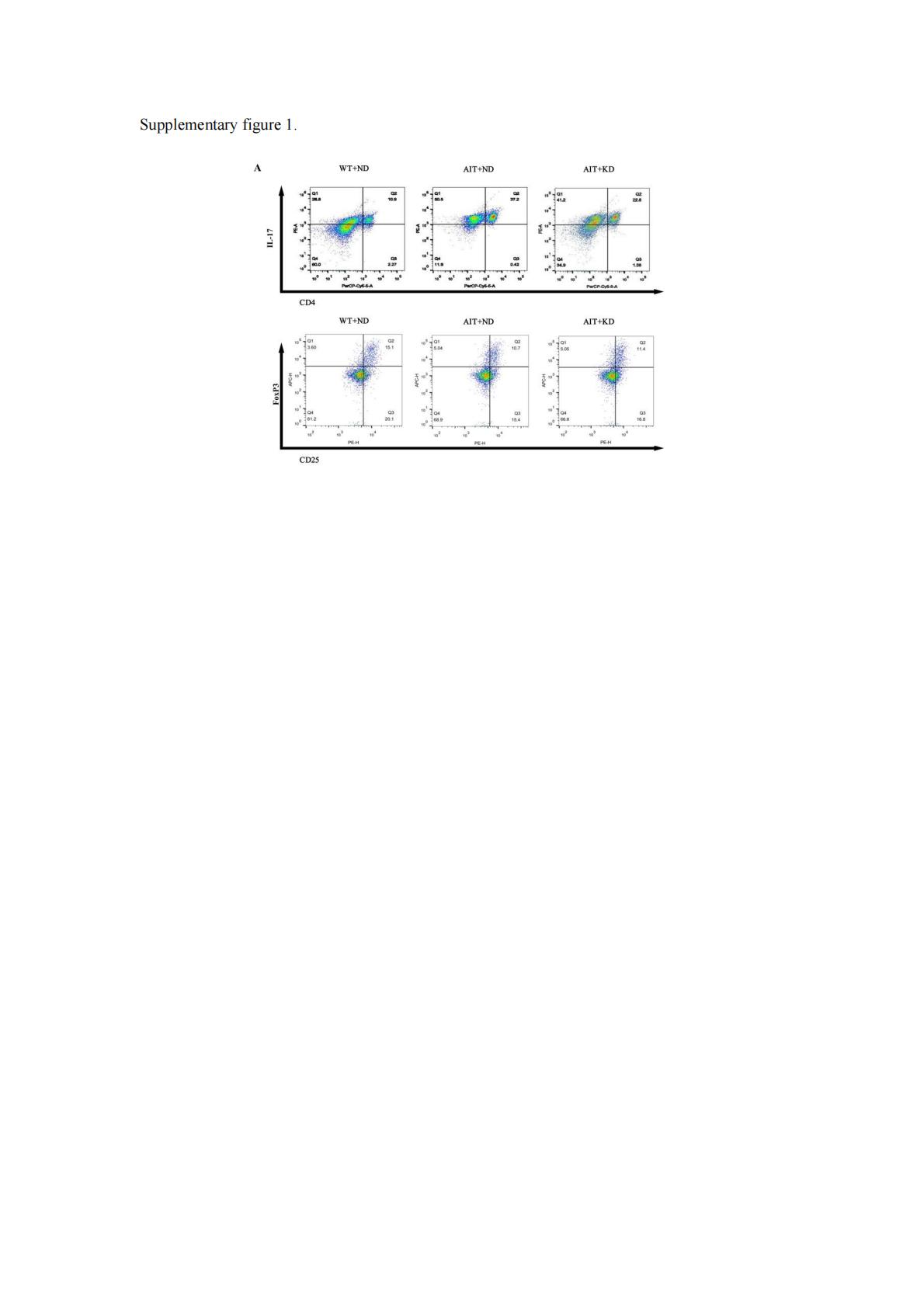

Supplement: S1 Fig — (A) Flow cytometry plots of Th17 cells in the spleens of mice in each group. (B) Flow cytometry plots of FoxP3 cells in the spleens of mice in each group. (JPG) [file pone.0341564.s006.jpg]
