# Supplementary material for: The ketogenic diet alleviates autoimmune thyroiditis caused by Th17/Treg imbalance by inhibiting the HMGB1/NLRP3 signaling pathway
Source: PLoS One. 2026 May 8;21(5):e0341564. doi: 10.1371/journal.pone.0341564 (PMC13155659; doi:10.1371/journal.pone.0341564)
Supplement: Attachment — Submitted filename: 2025-7-14-Response to reviewers.docx [file pone.0341564.s008.docx]

#### **Response to the Reviewer’s Comments**

**Manuscript number:** PONE-D-25-04384R1

**Title:** The Ketogenic Diet Alleviates Autoimmune Thyroiditis Caused by Th17/Treg Imbalance by Inhibiting the HMGB1/NLRP3 Signaling Pathway

Dear Editors and Reviewers,

We appreciate the opportunity to revise our manuscript titled "The Ketogenic Diet Alleviates Autoimmune Thyroiditis Caused by Th17/Treg Imbalance by Inhibiting the HMGB1/NLRP3 Signaling Pathway" and are grateful for the insightful comments provided by the reviewers. Those comments are all valuable and very helpful for revising and improving our paper, as well as the important guiding significance to our researches. In the following, we have provided detailed responses to each of the reviewers' comments. Revised portion are marked in red in the paper. We have tried our best to make all the revisions clear, and we hope that the revised manuscript meets the requirements for publication.

**Comment 1**: 1.Can you please upload an additional copy of your revised manuscript that does not contain any tracked changes or highlighting as your main article file. This will be used in the production process if your manuscript is accepted. Please amend the file type for the file showing your changes to Revised Manuscript w/tracked changes. Please follow this link for more information: http://blogs.PLOS.org/everyone/2011/05/10/how-to-submit-your-revised-manuscript/. Kindly check page 6 and the red citation for figure 6 on page 19.

**Response 1**: Thank you for your suggestion. We have uploaded the revised manuscript (marked-up copy) and the revised version (clean copy) , and highlighted page 6 and Figure 6 in the marked version of the revised version.

**Comment 2**: We note that your author list was updated during the revision process. In order to add or remove authors or update the order of the author byline after initial submission, we ask that authors complete an Authorship Change Request form. You may review our full authorship change policy and download the Authorship Change Request form here: <https://journals.plos.org/plosone/s/authorship#loc-authorship-changes.> Please fill out all required sections of the form. If you are adding or removing more than 2 authors, you can complete multiple forms and submit as many forms as needed to reflect the updates. Please return the form(s) as an attachment by emailing plosone@plos.org or by uploading it as a submission file labeled with the file type ‘Other’. The form will be reviewed by PLOS staff for approval. Should the changes be approved, we will require written confirmation from all authors confirming that they approve of the changes. Please note that if your manuscript is accepted, we will not be able to complete the publication process without the completed form.

**Response 2**: Thank you for your suggestion. After verification, this revision does not involve any changes to the author list (the original author team remains). We have resubmitted a cover letter, which contains the author's contribution statement.
