# Supplementary material for: The ketogenic diet alleviates autoimmune thyroiditis caused by Th17/Treg imbalance by inhibiting the HMGB1/NLRP3 signaling pathway
Source: PLoS One. 2026 May 8;21(5):e0341564. doi: 10.1371/journal.pone.0341564 (PMC13155659; doi:10.1371/journal.pone.0341564)
Supplement: Attachment — Submitted filename: 10-15Rebuttal Letter.docx [file pone.0341564.s009.docx]

#### **Response to the Reviewer’s Comments**

**Manuscript number:** PONE-D-25-04384R1

**Title:** The Ketogenic Diet Alleviates Autoimmune Thyroiditis Caused by Th17/Treg Imbalance by Inhibiting the HMGB1/NLRP3 Signaling Pathway

Dear Editors and Reviewers,

We appreciate the opportunity to revise our manuscript titled " The Ketogenic Diet Alleviates Autoimmune Thyroiditis Caused by Th17/Treg Imbalance by Inhibiting the HMGB1/NLRP3 Signaling Pathway" and are grateful for the insightful comments provided by the reviewers. Those comments are all valuable and very helpful for revising and improving our paper, as well as the important guiding significance to our researches. In the following, we have provided detailed responses to each of the reviewers' comments. Revised portion are marked in red in the paper. We have tried our best to make all the revisions clear, and we hope that the revised manuscript meets the requirements for publication.

Review：1.We note that there were multiple versions of Cover Letter, Manuscript, Response to Reviewers in your submission's file inventory. We have removed the older file(s), retaining the most recent version(s) for editorial review. Please double check your submission file inventory and let us know if any files appear to be outdated or absent.

Reply：We have already checked my submission file inventory and removed the older files.

Review 2.Please note that PLOS ONE requires submissions reporting blot or gel data to comply in full with the reporting requirements described at https://journals.plos.org/plosone/s/figures#loc-blot-and-gel-reporting-requirements. We now require authors to provide the original unadjusted and uncropped images for any blot or gel data reported in PLOS ONE submissions. In our internal checks for your submission, we noted that you did not provide original raw image files supporting blot/gel data in response to our previous request.

Reply: We have added gel data in your submission's file inventory named as “S1_raw_images”.
