# Supplementary material for: The ketogenic diet alleviates autoimmune thyroiditis caused by Th17/Treg imbalance by inhibiting the HMGB1/NLRP3 signaling pathway
Source: PLoS One. 2026 May 8;21(5):e0341564. doi: 10.1371/journal.pone.0341564 (PMC13155659; doi:10.1371/journal.pone.0341564)
Supplement: Attachment — Submitted filename: 2026-01-06-Response Letter.docx [file pone.0341564.s010.docx]

#### Response to the Reviewer’s Comments

**Manuscript number:** PONE-D-25-04384R2

**Title:** The Ketogenic Diet Alleviates Autoimmune Thyroiditis Caused by Th17/Treg Imbalance by Inhibiting the HMGB1/NLRP3 Signaling Pathway

Dear Editors and Reviewers,

We appreciate the opportunity to revise our manuscript titled"The Ketogenic Diet Alleviates Autoimmune Thyroiditis Caused by Th17/Treg Imbalance by Inhibiting the HMGB1/NLRP3 Signaling Pathway" and are grateful for the insightful comments provided by the reviewers. Those comments are all valuable and very helpful for revising and improving our paper, as well as the important guiding significance to our researches. In the following, we have provided detailed responses to each of the reviewers' comments. Revised portion are marked in red in the paper. Additionally, we have conducted a comprehensive revision of the entire manuscript. In this response letter, the reviewers' comments are presented in italics, and our corresponding changes and additions to the manuscript are highlighted in red text. We have tried our best to make all the revisions clear, and we hope that the revised manuscript meets the requirements for publication.

#### Reviewer Comments:

**REVIEWER #1.1: Mention the data analysis used. What was the percentage increase in TgAb/TPOAb levels, enhanced thyroid lymphocytic infiltration, elevated proinflammatory cytokines, and aggravated oxidative stress? What was the p-value for the difference?**

Reply 1: The tables below summarize the changes in TgAb levels, TPOAb levels, thyroid lymphocytic infiltration index, pro-inflammatory cytokines levels, and oxidative stress levels, respectively. (Page 12, line 311 and Page 13, line 327 and Page 13, line 335 )

Table 1 TgAb and TPOAb Levels

| Group | AIT+ND | AIT+KD | % | *P* value |
| --- | --- | --- | --- | --- |
| TGAb | 4.32 ± 0.31 | 3.36 ± 0.46 | 22.2 % | 0.0023 |
| TPOAb | 41.74 ± 15.13 | 24.63 ± 2.67 | 41.0 % | 0.0165 |
| Lymphocyte Infiltration Index | 3.20 ± 0.62 | 1.70 ± 0.47 | 46.9 % | <0.0001 |

Table 2 Proinflammatory Cytokines Levels

| Group | AIT+ND | AIT+KD | % | *P* value |
| --- | --- | --- | --- | --- |
| MCP-1 | 135.90±7.30 | 76.02±6.54 | 44.1 % | <0.0001 |
| TNF-ɑ | 500.1±59.02 | 396.9±23.64 | 20.6 % | 0.0023 |
| IL-1β | 53.80±6.30 | 44.04±6.30 | 18.1 % | 0.0224 |
| IL-6 | 62.20±10.51 | 47.99±5.29 | 22.9 % | 0.0105 |
| IL-18 | 53.17±6.75 | 44.13±7.06 | 17.0 % | 0.0486 |
| IFN-γ | 102.9±6.75 | 62.73±9.81 | 39.1 % | <0.0001 |

Table 3 Oxidative Stress Levels

| Group | AIT+ND | AIT+KD | % | *P* value |
| --- | --- | --- | --- | --- |
| MDA | 63.85 ± 6.13 | 50.00 ± 4.06 | 21.7 % | 0.0005 |
| SOD | 73.80 ± 8.26 | 97.90 ± 5.11 | 32.7 % | 0.0001 |
| T-AOC | 4.03 ± 1.26 | 9.69 ± 1.26 | 140.4 % | <0.0001 |

**REVIEWER #1.2: How many mice were used?**

Reply 1.2: Sixty 8-week-old SPF NOD.H-2h4 mice were stratified by body weight and then completely randomized into three experimental groups (n = 20 per group). This protocol satisfies the 3R principle while ensuring adequate statistical power.

**REVIEWER #1.3: Add a section at the end of the session for the strengths and weaknesses of the research.**

Reply 1.3: Using the international iodine-excess NOD.H-2^h4^ standard, we established a rigorous AIT model and provide the first integrated evidence that a ketogenic diet simultaneously suppresses the HMGB1–TLR/NF-κB–NLRP3 inflammasome axis and rebalances the Th17/Treg compartment, markedly attenuating thyroid damage and circulating autoantibody levels. These findings offer a novel dietary strategy for AIT management. Nevertheless, the present work is confined to in vivo proof-of-concept, and the precise molecular targets and regulatory hierarchies remain to be definitively elucidated in future studies. ((Page 20, line 536 )
